# Comparative Study of Volatile Compounds and Sensory Characteristics of Dalmatian Monovarietal Virgin Olive Oils

**DOI:** 10.3390/plants10101995

**Published:** 2021-09-23

**Authors:** Mirella Žanetić, Maja Jukić Špika, Mia Mirjana Ožić, Karolina Brkić Bubola

**Affiliations:** 1 Institute of Adriatic Crops and Karst Reclamation, Put Duilova 11, HR-21000 Split, Croatia; Maja.Jukic.Spika@krs.hr; 2 Centre of Excellence for Biodiversity and Molecular Plant Breeding (CoE CroP-BioDiv), Svetošimunska Cesta 25, HR-10000 Zagreb, Croatia; 3 Education and Teacher Training Agency, Tolstojeva 32, HR-21000 Split, Croatia; mmozic@azoo.hr; 4 Institute of Agriculture and Tourism, K. Huguesa 8, HR-52440 Poreč, Croatia; karolina@iptpo.hr

**Keywords:** virgin olive oil, *Olea europea* L., phenols, sensory profile, fatty acid composition, volatile compounds

## Abstract

Volatile compounds are chemical species responsible for the distinctive aroma of virgin olive oil. Monovarietal olive oils have a peculiar composition of volatiles, some of which are varietal descriptors. In this paper, the total phenolic content (TPC), fatty acid composition, volatile compounds, and sensory profile of monovarietal olive oils from four Dalmatian most common olive cultivars—Oblica, Lastovka, Levantinka, and Krvavica—were studied. The volatile composition of olive oils was analyzed using headspace solid-phase microextraction with gas chromatography/mass spectrometry. The highest mean TPC value was measured in Oblica and Krvavica oils (around 438 mg/kg). The difference among cultivars for fatty acids composition was detected for C16:1, C17:0, C18:1, C18:2, and the ratio C18:1/C18:2. Krvavica oils showed clear differences in fatty acid composition compared to oils from other cultivars. The most prevalent volatile compound in all oils was C6 aldehyde E-2-hexenal, with the highest value detected in Levantinka oils (75.89%), followed by Lastovka (55.27%) and Oblica (54.86%). Oblica oils had the highest value of Z-3-hexen-1-ol, which influenced its characteristic banana fruitiness, detected only in this oil. Lastovka oils had the highest amount of several volatiles (heptanal, Z-2-heptenal, hexanal, hexyl acetate), with a unique woody sensation and the highest astringency among all studied cultivars. Levantinka oils had the highest level of almond fruitiness, while Krvavica oils had the highest level of grass fruitiness.

## 1. Introduction

Food selection generally requires fulfilling fundamental nutritional needs: meeting basic nutrient content, improving physical and mental health, and reducing the risk of common diseases. Virgin olive oil (VOO) claims all of these features [1]. In addition, the food must also be delicious in order to be appealing to the consumer. The pleasant taste and aroma of VOO are the main attributes that guide consumers to choose this product among other vegetable oils. As a unique fat ingredient of the Mediterranean diet, VOO is well known as a vegetable oil produced directly from fresh olive fruit, processed mechanically under a strictly controlled temperature regime; it is consumed in an unrefined state [2]. For this reason, VOO keeps its original composition very similar to the one present in olive fruit while still being kept inside vacuoles. Its nutraceutical effect is due to its particular chemical composition since it consists of mainly a monounsaturated fatty acid (MUFA), namely, oleic acid (which controls cholesterol levels), and essential fatty acids, namely, linoleic and α-linolenic acids (which lower the risk of coronary diseases and the incidence of different types of cancers) [3]. Oxidation of VOO takes place in unsaturated fatty acids, so the main substrates in the olive oil’s glyceride part are oleic acid (about 55–83%) [4] and double unsaturated linoleic acid (3.5–21%), while triple unsaturated linolenic acid is present with less than 1% [4,5]. International standards [4,5] establish the limits for the purity criteria of olive oils and olive pomace oils, and the proportions of single fatty acids are one of the most important criterion parameters. Both mentioned regulations have the limit values for VOO categorization that are mutually harmonized. Furthermore, VOO is rich in valuable amounts of important bioactive compounds, mainly tocopherols, phenols, sterols, hydrocarbons, and carotenoids [3,6]. Many of these compounds are natural antioxidants that have a protective role against oxidative deprivation during oil storage as well as guard the human body against different diseases [6]. Moreover, phenolic compounds are responsible for the bitterness and pungency sensation, two positive and desirable sensory attributes in VOO. The chemical composition of monovarietal VOO intensely depends on agronomic factors and the olive cultivar (genetic origin) and also defines its volatile profile [6,7,8,9,10,11,12]. For the unique VOO aroma, the volatile compounds formed during the crushing of olives, the malaxation of olive paste, and after the extracting process of oil are principally responsible. This group of compounds involves mainly C5 and C6 units formed from polyunsaturated fatty acids during the enzymatic process of the lipoxygenase pathway [13,14] and other consequent bioprocesses through a technological oil extraction process. There is a positive correlation between the concentration of phenols and volatile compounds produced by LOX pathways and the intensity of single sensory properties [15]. This mechanism shows a strong link between essential fatty acids and volatile compounds that are formed via the enzymatic activity of lipoxygenase, hydroperoxydelyase, and other enzymes involved in this reaction. The synthesis of VOO aroma compounds is a subject of interest for many research groups [13,14,15,16].

The volatile fraction of VOO consists of five main groups of compounds: aldehydes, alcohols, esters, terpenes, and organic acids. The most common VOO volatile compounds are: C6 aldehydes (hexanal, *Z*-3-hexenal, *E*-2-hexenal), representing 60–80%; C6 alcohols (hexanol, *Z*-3-hexenol, *E*-2-hexenol); and C6 esters (hexyl acetate, *Z*-3-hexenil acetate) [10]. The volatile fraction has a particular composition regarding olive cultivars and can be used for the characterization of monovarietal VOOs [17,18,19,20,21,22,23,24]. Olive (*Olea europaea* L.) cultivars present a high variability for VOO volatile compounds and, similarly, of the aroma quality. This natural variation of aroma profiling is a result of the high olive genetic diversity [25,26,27], but also it depends on other factors such is fruit ripening degree [24,28,29], irrigation regime in the orchard [30], fruit processing conditions during oil extraction [31,32], and correct olive oil storage practice [33,34,35,36]. Different pre- and post-harvest factors influence the olive oil composition and quality of olive fruit [37]. The olive cultivar influences the physico-chemical and sensorial properties of the oil. An important impact of olive cultivar is on volatile compounds [10] and fatty acid composition [38]. The olive oil flavor depends on its area of geographical origin because it is influenced by environmental conditions [10]. The growing area has impact on cis-3-hexenal, cis-3-hexenol, hexanal, hexanol, trans-2-hexenal, trans-3-hexenol and trans-2-hexenol [38]. The geographical cultivation area was also found to influence fatty acid composition [39]. At colder locations of higher altitude, both studied cultivars (Oblica and Leccino) had higher amounts of stearic, linoleic, and linolenic fatty acids as well as a higher proportion of phenolic compounds but lower amounts of oleic fatty acids. At warmer locations of lower altitude, both cultivars had oils with lower levels of fruitiness, bitterness, and pungency. During olive ripening, oleic and linoleic acids increase and palmitic acid decreases because of different enzymatic activities [40]. Olive fruit maturation degrees play a crucial role in chemical and sensory elements [41]. Olive ripeness affects the volatile compound concentrations [42]. During the early maturation stage, olive fruits show higher volatile compound concentrations compared with the late stage of maturation. Fatty acid concentration is affected by the ripening of the olive fruit, and its concentration differs depending upon the variety [37]. Water deficits in olive orchards usually affect the sensory properties of olive oil, mainly in the high intensity of bitterness [41]. Water stress reduces olive production by reducing the endogenous esterases in fruit, while more water availability decreases volatile compounds (trans-2-hexenal, cis-3-hexen-1-ol, and hexanol) in the fruit [37]. Prolonged olive fruit storage between harvest and processing has a negative effect on the produced olive oil because of the possible hydrolyses of triglycerides to free fatty acids by lipase action. For this reason, it is advisable to process olive fruits as soon as possible (within 24 h after harvesting) [36]. The olive technology process has a strong influence on the overall quality of the obtained olive oil. The crushing methods influence the sensory element of olive oil [41]. Prolonged crushing time may degrade the quality of the olive oil. The milling system has an impact on oil quality as well as the sensory properties [37]. Prolonged malaxation generates higher temperatures of olive paste that negatively affect the quality and sensory characteristics of olive oil [37]. The malaxation process generates the olive oil aroma if the concentration of oxygen and phenolic compounds decreases due to the enzymatic oxidation of polyphenol oxidase and peroxidase [31]. Nowadays, modern centrifuge systems are equipped with two- or three-phase extractors. Two-phase systems reduce or eliminate the use of water in the process, with the double advantage of limiting the use of water and reducing or eliminating the production of wastewater. On the contrary, three-phase systems use a certain quantity of water to adjust olive paste density and ease oil extraction; in contrast, there is a significant loss of phenolic compounds with vegetable water [36]. Systems with two and a half phases are the most recent type of decanter that requires the addition of a reduced quantity of water and separates three fractions (oil must, wastewater, and wet pomace). The advantage of this system is a small quantity of wastewater and a greater quantity of phenolic compounds that remains in the oil [36]. Each of the olive processing technological systems comprises a temperature under 27 °C in order to retain olive oil quality and chemical composition as well as sensory characteristics. The proper storage of freshly produced high-quality extra virgin olive oil is indispensable in order to keep its peculiar characteristics for a longer period. The filtration method, light exposure to light, contact with oxygen or higher temperatures, and the trace elements that promote lipid oxidation also shorten the shelf life and sensory properties of olive oil [37]. Volatile compounds from VOO are also influenced by heating during cooking [43]. Comparing volatile profiles of extra virgin olive oil, olive pomace oil, soybean oil, and palm oil in different heating conditions, Giuffre et al. [43] found that heating completely changed the volatile organic compound content of all four studied edible vegetable oils. Extra virgin olive oils showed the highest number of components, with E−2-hexenal as the highest in quantity in fresh oil and Z-2-decenal and 2-undecanal as the highest in quantity in heated extra virgin olive oils. By the results of this study, the consumer can decide what temperature and heating time to apply in order to preserve the flavors and to reduce off-flavors that are produced during the heating of extra virgin olive oils and other studied edible oils [43]. The optimization of analytical methods applied for the determination of volatile composition is also a very important step that influences the aroma fingerprint of certain monovarietal VOOs [42,44,45,46,47,48,49]. The principal sensory characteristics appraised during the organoleptic assessment of VOOs are peculiar flavor features that are generated from volatile and phenolic compounds [50]. In the recent decade, particular research attention has been given to investigate the relationship between aroma compounds and specific VOO attributes perceived by panel groups, including the development of electronic noses [48].

In a recently published review [47], it was emphasized that, today, there are around 700 detected volatile compounds that contribute to the unique ‘green and fruity’ aroma of VOOs. Many authors have studied the relationship between sensory evaluation and the volatile profile of VOOs [49,51,52,53], analyzing the composition of aroma compounds responsible for both positive and negative sensory attributes in VOOs. The association of positive sensory properties with the presence of certain volatile compounds by chemometric approach in Italian VOOs confirmed the presence of 16 volatile compounds, 8 of which affect the green sensation, with the remaining 8 affecting sweetness [51]. Positive sensory characteristics were associated with C5 and C6 compounds, and the most abundant ones contributing positively to the aroma profile of VOOs are C5 and C6 aldehydes and alcohols [51].

Analyzing Italian and Spanish monovarietal VOOs, correlations were detected between the major volatile compounds (sum of aldehydes C6) and the orthonasal perception of olive fruitiness and the retronasal odor of almond [52]. Although modern analytical methods are needed to achieve the correct classification of olive oil, it is inevitable to connect the analytical detection based on the identification and quantification of volatile constituents of individual components and the results of panel group sensory evaluation. The latter is due to the complexity of education, training process, and skill [52].

Consumer ability to recognize the positive and negative sensorial attributes of VOOs that are related to their composition has been the research focus of different research groups [47,53,54,55]. Barbieri et al. [53] studied the ability of average consumers to distinguish olive oils by sensory properties that scientists label as ‘healthier’. However, this research has shown their greater susceptibility for ‘sweet’ aromas versus ‘bitter’ ones that assume health impact, which indicates an existing space and need for continuous consumer education. Besides positive sensory attributes, the presence of which are obligatory in VOOs, there is always a possibility that some undesirable negative attribute appears due to mistakes in fruit conservation before processing, inadequate oil processing, or/and storage conditions [50].

Croatia has a century-long olive growing tradition, and today, there are more than 40 autochthonous olive cultivars grown mainly in extensive olive orchards [54,56]. Although the production of olive oil is rather modest and represented mainly by small family olive farms, Croatian olive oils are becoming more and more eminent in the world due to their high quality and numerous awards at various international competitions. Olive and olive oil production in Croatia is constantly increasing. According to IOC data from 2012 [57], annual olive oil production in Croatia is about 5500 t, while table olive production is about 1500 t. According to the Central Bureau of Statistics, in 2017, in the Republic of Croatia, 28,947 tons of olives were produced and about 37,463 hL of olive oil. According to the same data, the area under olive groves in 2017 was 18,683 ha, with approximately 5.5 million olive trees [58]. The largest areas under olive trees are located in Split-Dalmatia County. About 50% of production is domestic autochthonous varieties. The predominant olive cultivar is Oblica, the autochthonous olive variety that covers about 75% of all olive orchards [58].

Although the research on Croatian olive oils done so far has been focused mainly on oil characterization based on fatty acid composition, phenols, volatile compounds, sterols, and sensory quality [29,32,59,60,61,62], the Dalmatian monovarietal VOOs’ volatiles and their sensory profiles have not been sufficiently studied. Dalmatia is a coastal part of Croatia, and it has traditionally been an olive-growing region for more than 2000 years [32]. The knowledge of the particular sensorial characteristics of monovarietal Dalmatian olive oils is very important for a better understanding of their specificity, which contributes to the creation of blends with intended sensorial profiles. In this paper, we study the composition of volatile compounds and the sensory characteristics of monovarietal VOOs obtained from the three most represented autochthonous Dalmatian olive cultivars: Oblica, Lastovka, Levantinka, and the lesser-known cultivar Krvavica; some of them are poorly investigated. Moreover, fatty acid composition and total phenol content of analyzed monovarietal VOOs were observed in order to find existing links between them and aroma volatile compounds. Biodiversity evaluation and the preservation of autochthonous olive cultivars are our focus points, with the principal aim of appraisal and raising the value of Croatian monovarietal VOOs.

## 2. Materials and Methods

### 2.1. Harvesting and Olive Oil Extraction

Healthy olive fruits from four autochthonous Croatian cultivars typical for the Dalmatian region (Oblica, Lastovka, Levantinka, Krvavica) were harvested manually during the second half of October 2012 (three trees per cultivar) from genetically identified cultivars. The olive trees were all cultivated in the same experimental field of the Institute for Adriatic Crops (Kaštela, Croatia) under the same pedoclimatic and agronomic conditions. The field is located 0.5 km from the coast (43_550 N; 16_350 E) and 28 m above sea level. It is influenced by the Mediterranean climate, defined as the Csa climate type [63]. The land there is an almost flat coastal plain, and the effective soil depth is 75 cm. It is clay-loam with an alkaline reaction, with a low-to-medium level of skeleton [39]. This experimental field is 70 years old, where non-irrigated olives grow. Other cultivation practices such as plant protection, pruning, and fertilization were applied in a manner consistent with accepted commercial practices. Oblica has medium vigorous trees with a rounded canopy. Its fruits are spherical-shaped, with an average weight of 5 g and 18–21% oil yield [56]. Due to uneven ripening during the harvest period, the fruits are different colored, from green to dark purple. Lastovka has a medium lush pyramidal canopy with a short and very forked trunk. Its fruits are elliptic-shaped, with an average weight of 3 g and about 24% oil yield [56]. Lastovka has a late ripening period, and the fruit is completely black at the fully mature stage. Levantinka has a very dense rounded canopy with an elegant and smooth trunk. Its fruits are medium-sized and formed in clusters, elliptically elongated, and slightly curled towards the top. The medium fruit weight is about 4.5 g with a 20% oil content [56]. Levantinka has a medium ripening period. Krvavica has a very vigorous canopy and a strong trunk. Its fruit is spherical-shaped and slightly elongated towards the top, with an average fruit weight of 3.5 g and an 18% oil yield [56]. Krvavica has a medium ripening period, with dark purple to black colored completely ripe fruits. All fruits were harvested with a similar fruit maturity index (MI = 1.5–2.0), which is calculated using fruit skin and pulp coloration grades [26]. The fruits collected from each tree, as three biological replicates, were processed separately into oil (for each cultivar, three trees with corresponding three oil samples). The fruits were processed within 24 h from harvesting using centrifugal extraction in two phases on a laboratory small-scale Abencor system (MC2 Ingeniería y Sistemas S.L., Sevilla, Spain) that simulates industrial processing with controlled producing parameters. Approximately 1 kg of fruits was crushed using a hammer mill (MM-100) with an easily detachable stainless-steel hammer of 4.5 mm diameter. The obtained olive paste was malaxed in a thermomix bath (TB-100) for 35 min under a strictly controlled temperature (26 ± 1 °C). After malaxation, the prepared olive paste was immediately subjected to centrifugation using a centrifugal machine (CF-100) with a rotation speed of 3500 rpm and a duration of 90 s. The obtained olive oil samples were clarified by additional fine centrifugation using a Hettich Universal 320R centrifuge machine (Andreas Hettich GmbH & Co. KG, Tuttlingen, Germany) with a cooling system at 18 °C and a speed of 5000 rpm for 4 min. All samples were stored in dark glass bottles under the atmosphere of nitrogen at a temperature of −20 °C for one month until analyses.

### 2.2. Olive Oil Qualitative Parameters

In all obtained olive oil samples, the basic quality parameters (FFA—free fatty acids, expressed % of oleic acid; PV—peroxide value, expressed as meq O_2_/kg; and UV spectrophotometric indices at 232 and 270 nm—K_232_ and K_270_) were analyzed according to EEC methodology [4]. All results are expressed as mean values of three measurements. All chemicals and reagents were acquired from Carlo Erba Reagents (Val de Reuil Cedex, France).

### 2.3. Determination of Total Phenolic Content

Total phenolic content (TPC) in investigated VOOs was determined by the spectrophotometric method [64]. The separation of phenolic extracts was performed as follows: the liquid–liquid extraction of the previous prepared hexane/oil solution with a water/methanol mixture (60:40, *w*/*w*) was used three times in a row. The Folin-Ciocalteu reagent (Sigma-Aldrich, St. Louis, MO, USA) was used for the colorimetric reaction. The measurements of absorbances were performed in triplicate at 765 nm on the Cary 50 UV–vis spectrophotometer (Varian, CA, USA), and the results are expressed as mean values and presented as mg of Gallic acid per kg of oil.

### 2.4. Determination of Fatty Acid Composition

Fatty acid methyl esters (FAME) from VOO samples were obtained by alkaline treatment with 1M KOH in methanol using capillary column DB-WAX (film 0.25 μm; 30 m × 0.25 mm; Agilent, Santa Clara CA, USA), as described in ISO method [65]. The profiles of fatty acids in monovarietal VOOs were determined by gas chromatography separation of prepared methyl esters, according to the ISO method [66]. The results are expressed as average from three replicates for each sample.

### 2.5. Analysis of Volatile Composition by HS-SPME/GC-MS

The volatile compounds of VOOs were extracted using headspace solid-phase microextraction (HS-SPME) and analyzed by gas chromatography/mass spectrometry (GC-MS) using a Varian 3900 GC instrument coupled to a Varian Saturn 2100T ion trap mass spectrometer (Varian Inc., Harbur City, CA, USA) according to slightly modified methodology, as described by Brkić Bubola et al. [12]. Differently from the above-cited method, a VOO sample (3.5 g) was placed in a 10 mL vial. The identification of volatile compounds was performed by comparing their mass spectra with those of pure standards and to mass spectra from the NIST05 library. Additionally, the identification of 10 volatile compounds was carried out by comparing their retention times with those of pure standards. All standards had a GC purity ≥95% and were purchased from Aldrich (Steinheim, Germany) and Fluka (Buchs, Germany). Moreover, Kováts’ retention indices (KIs) were determined on a polar Rtx-WAX capillary column (Restek, Bellefonte, PA, USA) by injection of a standard mixture containing the homologous series of normal alkanes (C6-C24) in pure dichloromethane and compared with the retention indices of the compounds available in the literature [18,23,38,48,67]. The relative proportions of the volatile compounds were obtained by peak area normalization. For each volatile compound, the mean proportions of three independent repetitions are reported.

### 2.6. Sensory Analyses of VOOs

A trained professional panel of the Institute for Adriatic Crops from Split, Croatia, approved by the Croatian Ministry of Agriculture, performed a sensory evaluation of monovarietal VOOs using the official IOC method [68]. All panel members conducted training according to the standard IOC method [69] using reference samples obtained directly from IOC Madrid, Spain. All oil samples were evaluated by 8 tasters. The descriptive sensory analysis was used for a detailed description of each monovarietal VOO, and positive and negative sensory attributes were perceived independently by each panel member. The IOC profile sheet used for this evaluation was expanded with positive descriptors listed in the IOC methods for sensory assessment of extra virgin olive oils with Protected Designations of Origin (PDOs) [70]. The intensity of each descriptor was graded individually by tasters using a continuous unstructured scale of 10 cm. The results are presented as the median values of the tasters’ sensory perceptions [68].

### 2.7. Statistical Analyses

The results of all chemical analyses obtained with this investigation were subjected to a one-way analysis of variance (ANOVA). Firstly, the data were tested for normality and homogeneity of variance and transformed when necessary. Mean values were linked by Tukey’s honest significant difference test with the level of *p* ≤ 0.05. Statistical analysis was performed using Statistica v. 13.2 software (Stat-Soft Inc., Tulsa, OK, USA).

## 3. Results and Discussion

### 3.1. VOO Quality Assessment

The determination of basic quality parameters is one of the fundamental preconditions for the categorization of an olive oil. The values for FFAs, PVs, and specific coefficients of extinction at 232 and 270 nm (K_232_ and K_270_) and their respective ΔK values for investigated monovarietal virgin olive oil samples are presented in Table 1.

The acidity values ranged from 0.2% to 0.4%. Peroxide value, an indicator of primary auto-oxidation products, ranged from 3.7 (Krvavica) to 6.5 (Lastovka) meq O_2_/kg. Values of specific coefficients of extinction are measured at 232 (K_232_) and 270 nm (K_270_), corresponding to the maximum absorption of the conjugated dienes and trienes. They are formed during autoxidation from the hydroperoxides of unsaturated fatty acids and their fragmentation products [27]. Data for K_232_ and K_270_ did not show any differences among monovarietal oils. Comparing all these results with the limits set up by international regulation [4,5], it is evident that all investigated oils were in the category of extra virgin olive oil.

In addition, the values for TPC in monovarietal VOOs are presented in Table 1. Although TPC is not officially considered a quality indicator prescribed by regulation, it is a very important parameter related to the health properties of VOOs and their sensorial characteristics. The obtained results for TPC values in the four Dalmatian monovarietal olive oils indicated significant differences among the monovarietal olive oils. The highest TPC value was detected in the Krvavica oils, which did not differ from the TPC value for oils from Oblica. Oils from Lastovka and Levantinka had similar values for TPC. The mean TPC value in Oblica oil (438.3 mg/kg) were in concordance with the data obtained in the study of Jukić Špika et al. [39] for the same crop year, but still lower compared with another study of Lukić et al. [59] during the crop year of 2015, where the fruit ripening showed the strongest effect on the accumulation of phenolic compounds. Levantinka oils had a mean TPC value (302.1 mg/kg) higher than the detected value for this monovarietal oil in a previous study [61]. Additionally, the mean TPC values measured in Krvavica olive oils were much higher than those found in the literature [62]. On the contrary, in Lastovka oils, the mean TPC value (312.0 mg/kg) was slightly lower compared to the highest TPC values reported in previous research [61]. These statements could be related to different maturity indices of fruits and also to different technological processes since our samples were processed in a pilot-scale system. It is known that total phenolics content changes during the ripening period and depends on the crop year and also the cultivar [7,8,29,39]. In our study, the olive fruits of each cultivar had the same maturity index at the moment of harvesting (MI = 1.5–2.0) in order to decrease the possible influence of this factor on TPC in the analyzed olive oil samples.

### 3.2. Fatty Acid Composition 

Monovarietal olive oils differ in the fatty acids’ composition depending on the olive cultivar [50]. Different authors have studied the composition of fatty acids in order to characterize the olive cultivar [47,56,60]. The fatty acid composition of Dalmatian monovarietal olive oils is presented in Table 2. In all investigated oil samples, palmitic acid (C16:0) has been identified with a medium value of 12.58%. Palmitic acid (C16:0), the major saturated fatty acid in olive oil, had the highest proportion in Krvavica olive oils (about 14%), while the lowest content was detected in Levantinka oils (about 12%) even though no statistically significant difference in palmitic acid content among studied cultivars was determined. The significant highest value of palmitoleic acid (C16:1) was detected in Krvavica oils. Both heptadecanoic and heptadecenoic acids (C17:0 and C17:1, n-9) were detected in low amounts in all oil samples, with an average of 0.05% and 0.11%, respectively. Regarding heptadecanoic acid, the highest content was detected in Lastovka oils. Comparing our results with other studies, Italian cultivar Frantoio [71] had higher C17:1 values (0.20–0.30%) measured for different growing sites.

For all analyzed oils, the content of stearic acid (C18:0) had a medium value of 1.61%, with no significant differences detected among cultivars. All analyzed oil samples had contents of monounsaturated oleic acid (C18:1) higher than 70%. The significant difference for C18:1 was observed in Lastovka oils, which had the lowest value. At the same time, this oil had the highest content of linoleic (C18:2) compared to other studied monovarietal oils. In Oblica VOOs, the mean value of C18:2 was 9.66%, followed by Levantinka with 6.78%, while the significantly lowest content of linoleic fatty acid was detected in Krvavica olive oils (4.62%). In comparison with data from another study [39], Oblica had a high C16:0 content (average 13.43%) as well as a medium content of C18:2 (average 11.22%). C18:2 content in the Leccino cultivar grown in Croatia [39] was of an average of 6.99% (5.53–9.74%), while Frantoio oils had C18:2 contents in the range of 6.20—8.20% (average 7.38%) [71].

There was no significant difference among other less represented fatty acids (arachidonic, gadoleic, behenic, and lignoceric acid) identified in the obtained VOOs (Table 2). Based on EU regulation [4], all analyzed monovarietal olive oils were placed in the category of extra virgin olive oil, according to fatty acid composition. It was evident that the Krvavica cultivar showed clear differences in fatty acid composition compared to oils from other cultivars. Therefore, this feature could be considered a characterization point for Krvavica olive oils, as it was highlighted in another study that fatty acid composition can be used as a base for the characterization and evaluation of VOOs [62]. Different studies [40,61,72] have investigated the association between oleic and linoleic acids (18:1/18:2) and the oxidative stability of VOO. The results showed that the ratio of monounsaturated/polyunsaturated fatty acids is one of the key factors responsible for evaluating the oxidative stability of VOOs, and it is used as a parameter for oil characterization [73]. In our study, a significant difference for the C18:1/C18:2 ratio was observed among studied cultivars. Oblica and Lastovka oils showed a stable index expressed by the ratio of oleic acid to linoleic acid, with values near or higher than 7, while Levantinka and Krvavica oils had higher medium ratios of oleic acid/linoleic acid (11.24% and 16.83%, respectively), which implicate their high stability and resistance to oxidative degradation and, consequently, longer shelf life under correct storage conditions. The same conclusion was confirmed by the ratios of MUFA/PUFA (Table 2). Since VOO consists mainly of monounsaturated fatty acid (C18:1, MUFA) and significant amounts of polyunsaturated fatty acids (C18:2, C18:3, PUFA), it is considered a unique oil among other vegetable oils. There is an EFSA-approved health claim [74] on the unsaturated fatty acids, saying: ‘Replacing saturated fats in the diet with unsaturated fats contributes to the maintenance of normal blood cholesterol levels’. The claim may be used only for food that is high in unsaturated fatty acids, and VOO is, for sure, one of them.

### 3.3. Volatile Profiling of Dalmatian Monovarietal VOOs

**Figure 1:**
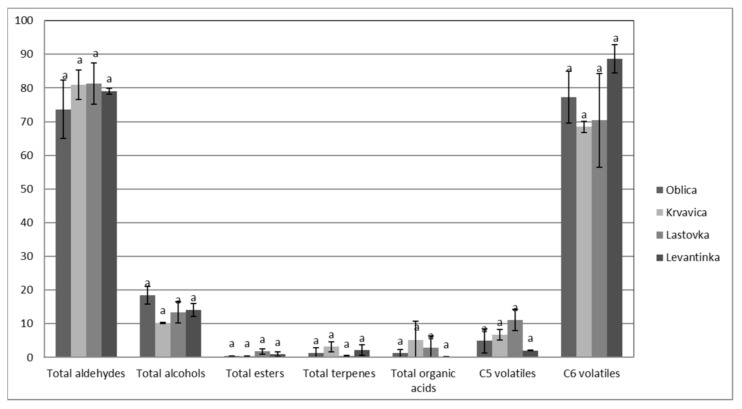
The amounts of volatile compounds (percentage of total peak area) in VOOs from Dalmatian autochthonous cultivars (results are expressed as means of 3 independent repetitions). The means labeled by different letters are significantly different (Tukey’s test, *p* ≤ 0.05).

The results of the volatile composition of the analyzed monovarietal olive oils from the four Dalmatian autochthonous cultivars are presented in Table 3. In total, 21 volatile compounds were detected, mainly aldehydes, alcohols, esters, terpenes, and organic acids. The same number of volatile compounds (21) was found in south Italian extra virgin olive oils in the study of Giuffrè et al. [43]. Different origin olive oils were studied by Luna et al. [50], and they found that Spanish and Italian VOOs had the highest values of total volatiles, with concentrations ranging from 19.0 to 27.0 mg/kg. Coratina was the variety with the highest value and Frantoio with the lowest one. The Spanish oils showed the widest concentration range (9.83–32.9 mg/kg), Nevado Azul being the variety with the highest value and Hojiblanca with the lowest one. With respect to Greek virgin olive oils, their concentrations ranged from 10.7 to 21.2 mg/kg. Finally, the non-European samples showed a total content of volatiles below 20 mg/kg, with the exception of Chemlal of Kabylie [50]. In our paper, even though no significant differences between monovarietal oils in total aldehydes, alcohols, esters, terpenes, and organic acids, as well as total C5 and C6 volatiles, were detected (Figure 1), some particularities in the volatile compound composition of monovarietal olive oils were found (Table 3).

In general, the most prevalent volatile compound in all oil samples was C6 aldehyde *E*-2-hexenal (Table 3), which contributes to green, fruity, bitter, and astringent sensory characteristics [48] and also bitter almond and green grass notes [51]. The same findings were presented in a study by Giuffrè et al. [43], where E−2-hexenal was found at an average of 28.3% in all analyzed south Italian extra virgin olive oils. The highest value for *E*-2-hexenal in our study was detected in Levantinka oils (74.84% of total peak area), while the other three monovarietal olive oils had similar values of *E*-2-hexenal. Significant differences among cultivars were found in certain aldehydes, namely, hexanal, heptanal, and *Z*-2-heptenal (Table 3). Lastovka oils had the highest heptanal and *Z*-2-heptenal amounts. The same monovarietal oil had the highest amount of hexanal (Table 3), C6 aldehyde is associated with the sensory characteristics of green fruitiness, apple, and green grass [48], while the other cultivars had a lower amount. Levantinka oil had the lowest amount of hexanal (1.34% of total peak area). In our study, Krvavica oils had a higher hexanal content than those found in an earlier investigation [62], probably due to a higher maturity index (MI = 4.3) than in our study (MI = 1.5–2). Hexanal, *E*-2-hexenal, and *Z*-3-hexenal are C6 aldehydes that arise during the malaxation of olive paste, and they occur in a series of enzymatic reactions known as the lipoxygenase pathway (LOX), from linoleic or alpha linolenic acids with lipoxygenase-catalyzed oxidation [50]. Hydroperoxide lyase is quite dependent on the variety, so its activity and concentration lead to different volatile profiles and, thus, the sensory properties of monovarietal oils [17], as confirmed in literature [59]. 

The C5 and C6 volatile contents mainly depend on genetic origin, which is responsible for enzymatic expression, and agronomic and technological parameters that strongly influence enzymatic activity [10]. The same group of volatile compounds are also present in different fruits and vegetables [50]; thus, it is understood why some olfactory sensations in VOOs are described as fruity sensations of apple, banana, almond, or artichoke.

Alcohols in VOOs are associated with positive sensory characteristics, such as green, fruity, bitter, and aromatic, although they have weaker sensory importance than aldehydes [48]. The highest, although not significantly, total alcohols were detected in Oblica oils (Table 3), mainly due to the highest values of *Z*-3-hexen-1-ol (13.25% of total peak area) (Table 3), which is related to the banana fruity sensation [37,50]. These results support the banana fruitiness of Oblica VOO as a cultivar descriptor, as declared in a previous paper [49] and confirmed in our study as well (Figure 1). Other alcohols were found to be prevalent in Levantinka (hexanol) and Lastovka (*E*-2-penten-1-ol) VOOs.

Esters detected in analyzed olive oils had low values in general (Table 3). This group of compounds is related to sweet and fruity sensory attributes [37,55], and significant differences among cultivars were not found except in the case of hexyl acetate. The highest value for this ester was measured in Lastovka oils (0.63% of total peak area), while it was not detected in Oblica oils. C6 esters have synergistic interactions with other aromatic compounds [46].

Regarding the terpenes, only one was detected in Dalmatian olive oils: α-copaene. No significant difference was found among monovarietal oils in α-copaene (Table 3), but in Krvavica oils, higher values were detected compared to data in a previous study [62].

**Figure 2:**
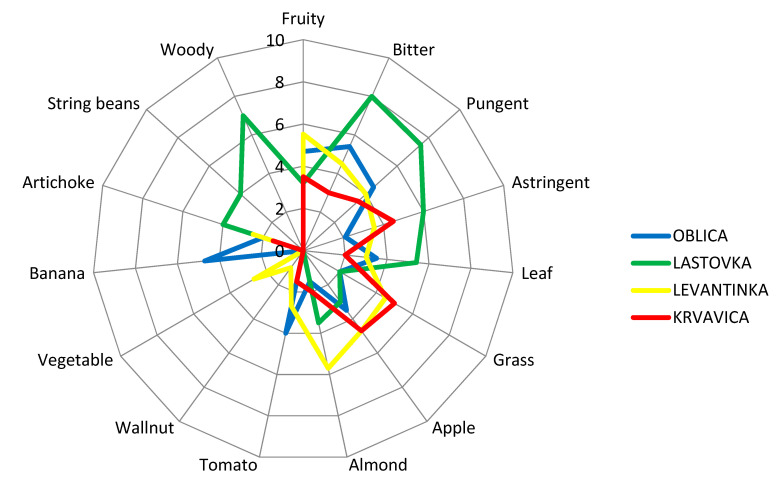
Sensory profiles of virgin olive oils obtained from four Dalmatian autochthonous cultivars. Results are expressed as the mean value of medians of three repetitions for each descriptor.

The presence of acetic and propanoic acids indicates the possibility of microbial fruit fermentation or some other incorrect step during fruit handling and is linked to sour and pungent attributes [37,50]. These acids also can generate negative sensory defects of winey–vinegary taste in VOOs [50]. In our study, total organic acids had very low values in all analyzed olive oils (Table 3), which supported the results of the sensorial analysis, where no negative attributes were detected (Figure 2).

### 3.4. Sensory Characteristics of Monovarietal VOOs

Sensory analyses of VOOs are a crucial step in the determination of the olive oil category, according to EU legislation [4,68]. Currently, sensory assessment by a well-trained panel group of experts remains an inevitable instrument in the organoleptic appraisal and aroma recognition of VOOs. During sensory analyses of VOOs, according to IOC methodology [68], the positive and negative attributes are perceived by smelling and tasting the olive oil sample. Besides the fruitiness that is inevitable for the category of VOO, bitterness and pungency are very desirable features of VOO, although those characteristics are not reflected as important in commodity classification [75]. The attributes of bitter and spicy oil are due to the presence of phenolic compounds. The bitter taste is attributed to compounds of the aglycone and dialdehydic forms of decarboxymethyl oleuropein and other forms of oleuropein aglycone. The pungent note has been attributed to the aglycone form of the dialdehydic of decarboxymethyl ligstroside [37]. In order to provide a more detailed organoleptic description and create a sensory profile of studied monovarietal olive oils, quantitative descriptive sensory analysis was carried out by a professional sensory panel using the IOC methodology developed for sensory evaluation of olive oils with protected designations of origin (PDOs) [70]. The sensory descriptive profile of monovarietal VOOs from our study is presented in Figure 2. Oblica olive oils were characterized with typical fruity sensations of banana, tomato, and apple, with well-pronounced and equilibrated bitterness and pungency (Figure 2). Distinct banana fruitiness was detected only in Oblica oils. Astringency and an almond sensation had the lowest intensity in Oblica olive oils compared to oils from other olive varieties. A slight note of green grass and artichoke fruitiness was also perceived in Oblica oils. A grass sensation was of equal intensity in Oblica and Lastovka oils and lower by 2.25 times than its value in Levantinka and 2.5 times than in Krvavica oils. Among average consumers, Oblica is considered to be a rather sweet olive oil [61]. However, it is confirmed that in the right maturity moment, its oil has satisfactory and rounded bitterness and pungency [59], two positive attributes that are linked to the positive health impact of this oil due to the presence of phenolic compounds [76]. The sensory profile of Lastovka oils showed the highest astringency intensity compared to other studied cultivars: 3 times higher than in Oblica oils, 1.7 times higher than in Levantinka oils, and 1.33 times higher than in Krvavica oils. Lastovka olive oil was characterized by mild fruitiness, similar to green leaves and apple. No tomato fruitiness was detected, unlike oils from the other cultivars. In Lastovka olive oil, high intensity of a woody sensation was perceived (which could be explained by the high amount of hexanal found in these oils) (Table 3). Although the presence of hexanal is regularly linked to green/grassy aromas in olive oil [50], Mayuoni-Kirshinbaum and Porat [77] reported the possible connection between hexane and woody flavor in their review of the flavor of pomegranate fruit. A woody attribute was perceived only in Lastovka oils, the same as a string bean sensation. Literature findings have declared that certain volatile compounds, mainly from the sesquiterpenes group, could also be responsible for the woody sensory sensation in fruit [78]. This fact was confirmed by Lukić et al. [78] in the case of Lastovka olive oil, applying a combined targeted and untargeted profiling of volatile aroma compounds. There is a possibility that the woody aroma in Lastovka oils appeared due to the presence of hexanal [77] or some sesquiterpenes [78]. The almond fruitiness found in Lastovka oils could be linked to the presence of *Z*-2-penten-1-ol, which is positively related to this sensory note [78].

In Lastovka oils, which are characterized by the strongest bitterness, pungency, and astringency compared to olive oils obtained from other cultivars, the high value of ethyl acetate and 1-penten-3-ol (Table 3) could have an influence on the taste characteristics since these volatiles are correlated with the ‘taste’ notes of pungency and astringency [2,37]. The presence of ethyl acetate and 1-penten-3-ol may have contributed to the astringency in Lastovka oils, in addition to the phenolics found in the oil, although not present in high amounts. Research was conducted on regular Italian consumers of olive oil who were familiar with olive oil on a daily basis, and the taste–smell interaction between bitterness and green grass odors was investigated [79]. The results confirmed that the green odor note had a significant positive effect on consumers’ perception of bitterness, which increased in the presence of green grass odors [79]. In our study, that could be the case for Levantinka oils, where the highest amount of *E*-2-hexenal was detected (Table 3), which could enhance the bitterness sensation of this oil. Additionally, the bitterness and grass sensations were equally graded in this oil (Figure 2). Moreover, Levantinka oils were characterized with the highest level of almond fruitiness compared with olive oils from the other three studied cultivars, accompanied with apple nuances, which was confirmed by the highest value for *E*-2-hexenal among all investigated monovarietal oils (Table 3), while tomato and vegetable notes were less graded. A slight note of walnut was detected only in Levantinka olive oils. This oil had very well-balanced bitterness and pungency, which could be related to the presence of a green grass odor, as stated in literature [79]. Mild to medium fruitiness with grass and ripe apple notes were the olfactory characteristics of Krvavica VOOs. This cultivar could be a preferable choice for the majority of consumers who choose milder and sweeter olive oils compared to bitter and pungent ones [53]. Compared to other olive oils in this study, Krvavica oils had the highest level of grass fruitiness and the lowest level of artichoke attributes. The bitterness and pungency in Krvavica oils had the lowest intensity among all studied cultivars but were still delicate and well balanced, in addition to astringency, although the TPC content in this oil had a relatively high mean value (Table 1).

Caporale et al. [79] classified olive oil bitterness according to TPC values into four categories: unnoticeable bitterness (equal or lower than 220 mg/kg), slight bitterness (220–340 mg/kg), bitter oils (340–410 mg/kg), and quite bitter or very bitter oils (higher than 410 mg/kg). Considering this grouping, it can be concluded that in our investigation, two monovarietal VOOs can be placed in the second group as slight bitter oils (Lastovka and Levantinka), and two other oils (Oblica and Krvavica) belong in the fourth group as quite bitter oils. The sensory profiles of the studied monovarietal Dalmatian olive oils (Figure 2) do not follow this grouping in full. VOO aroma is the effect of complex reactions inside the product (enzymatic, chemical, and so forth) that lead to the formation of volatile compounds [1]. Positive and negative synergisms can appear, and this could result in new types of perceptions and interactions between taste and odor [1]. Considering this claim, it can be concluded that besides the phenolics present in olive oils, volatile compounds play an important role or have a synergistic effect on certain sensory attributes of olive oil. The phenolic compounds were attested to have an important role in the intensity and timing of the release of certain aroma compounds during the consumption of virgin olive oil. During sensory analyses, high levels of VOO phenolic compounds resulted in a smaller total release of 1-penten-3-one, *E*-2-hexenal, and esters during the swallowing of the olive oil sample [80]. This fact could be explained by the formation of complexes between phenolic compounds and salivary proteins that capture aroma compounds and, therefore, decrease their volatility during the organoleptic assessment of olive oil. Since phenolic compounds can affect the release of VOO aroma compounds during its consumption, thereby influencing flavor perception and consumer acceptance, this interesting link should be further investigated in the case of monovarietal Croatian olive oils.

## 4. Conclusions

The natural variation and great variability of VOO volatile compounds offer a wide range of different flavor and aroma characteristics. Autochthonous olive cultivars provide important raw materials for the production of specific monovarietal olive oils that have the unique opportunity to gain high market positioning and obtain higher prices. In this paper, the chemical composition and sensory characteristics of Dalmatian monovarietal VOOs from a small-scale plant were compared and their peculiar characteristics demonstrated; on this basis, they can find their place in the demanding olive oil world market. Oblica olive oils, with typical banana fruitiness (present only in this cultivar oil and confirmed with the highest value of *Z*-3-hexen-1-ol) and apple and tomato notes, had clear and equilibrated bitterness and pungency. In this oil, a slight sensation of green grass and artichoke fruitiness was also detected. Olive oils from Lastovka had the highest amount of several volatiles (hexanal, heptanal, and *Z*-2-heptenal). Sensory attributes that confirmed this are mild fruitiness that recalls green leaves, almond, and apple, with a high intensity of a unique woody sensation, strong bitterness, pungency, and the highest astringency level among all studied cultivars. Levantinka olive oils had the highest amount of C6 aldehyde *E*-2-hexenal, the most prevalent volatile compound in all investigated oil samples. This oil had the highest level of almond fruitiness, with perceived apple notes, very well-balanced bitterness, and pungency. Krvavica olive oils differed from the analyzed monovarietal VOOs, particularly by fatty acid composition (highest proportion of palmitic acid, significantly highest value of palmitoleic acid, significantly lowest content of linoleic acid, and a high ratio between monounsaturated and polyunsaturated fatty acids). Olive oils from Krvavica had the highest level of grass fruitiness compared to other studied olive cultivars. In these oils, delicate bitterness and pungency were well balanced with astringency. The obtained results in this study contributed to the recognition of the specificity of Croatian monovarietal olive oils, which could contribute to the protection and preservation of the national Croatian olive genetic pool. The results of this study are also important data for producers to encourage them to create their own unique high-quality VOO based on domestic autochthonous olives.

## Figures and Tables

**Table 1:** Quality parameters of monovarietal virgin olive oils obtained from Dalmatian autochthonous cultivars.

	Cultivar				
Parameter	Oblica	Lastovka	Levantinka	Krvavica	EVOO *
FFA% (oleic acid)	0.4 ± 0.1 a	0.4 ± 0.0 a	0.4 ± 0.2 a	0.2 ± 0.0 a	≤0.8
PV (meq O_2_/kg)	3.7 ± 0.7 b	6.5 ± 0.1 a	6.3 ± 1.6 a	3.6 ± 0.2 b	≤20
K_232_	1.83 ± 0.31 a	1.74 ± 0.08 a	2.30 ± 0.19 a	1.85 ± 0.05 a	≤2.50
K_270_	0.16 ± 0.01 a	0.19 ± 0.03 a	0.16 ± 0.01 a	0.19 ± 0.01 a	≤0.22
∆K	−0.00 ± 0.00 a	0.00 ± 0.00 a	0.00 ± 0.00 a	−0.00 ± 0.00 a	≤0.01
TPC (mg Gallic acid/kg of oil)	438.3 ± 12.4 ab	312.0 ± 3.4 bc	302.1 ± 79.0 b	438.9 ± 52.0 a	

FFA—free fatty acid, PV—peroxide value, UV spectrophotometric indices (K_232_ and K_270_, ∆K), TPC—total phenol content, EVOO—extra virgin olive oil. Results are expressed as mean values of three repetitions ± standard deviation. The means within each row, labeled by different letters, are significantly different (Tukey’s test, *p* ≤ 0.05). * Actual limits for the EVOO category [4], except for TPC.

**Table 2:** Fatty acid composition of monovarietal virgin olive oils obtained from Dalmatian autochthonous cultivars.

Fatty Acid * (% of Total)	Cultivars				EVOO *
	Oblica	Lastovka	Levantinka	Krvavica	
C16:0	12.26 ± 0.38 a	12.32 ± 0.59 a	11.72 ± 0.13 a	14.00 ± 0.93 a	7.50–20.00
C16:1	0.75 ± 0.06 b	0.75 ± 0.04 b	1.04 ± 0.24 b	2.40 ± 0.43 a	0.30–3.50
C17:0	0.03 ± 0.01 b	0.10 ± 0.01 a	0.04 ± 0.00 b	0.04 ± 0.00 b	≤0.40
C17:1, n-9	0.08 ± 0.04 a	0.19 ± 0.06 a	0.08 ± 0.01 a	0.07 ± 0.00 a	≤0.60
C18:0	1.43 ± 0.51 a	1.71 ± 0.48 a	1.90 ± 0.10 a	1.40 ± 0.74 a	0.50–5.00
C18:1	75.91 ± 1.41 a	71.81 ± 1.15 b	76.17 ± 0.30 a	77.14 ± 0.34 a	55.00–83.00
C18:2	9.66 ± 0.85 b	11.63 ± 0.29 a	6.78 ± 0.19 c	4.62 ± 0.04 d	2.50–21.00
C18:3	0.66 ± 0.07 a	0.66 ± 0.04 a	0.66 ± 0.03 a	0.75 ± 0.15 a	≤1.00
C20:0	0.39 ± 0.06 a	0.48 ± 0.09 a	0.53 ± 0.11 a	0.30 ± 0.00 a	≤0.60
C20:1, n-9	0.36 ± 0.03 a	0.38 ± 0.11 a	0.40 ± 0.08 a	0.41 ± 0.00 a	≤0.50
C22:0	0.11 ± 0.01 a	0.10 ± 0.01 a	0.11 ± 0.01 a	0.10 ± 0.00 a	≤0.30
C24:0	0.10 ± 0.01 a	0.10 ± 0.00 a	0.10 ± 0.00 a	0.10 ± 0.00 a	≤0.20
C18:1/C18:2	7.92 ± 0.49 c	6.18 ± 0.09 d	11.24 ± 0.30 b	16.83 ± 0.53 a	-
MUFA	77.10 ± 1.47 ab	73.12 ± 1.35 b	77.68 ± 0.34 a	80.01 ± 0.76 a	-
PUFA	10.31 ± 0.92 a	12.28 ± 0.35 a	7.44 ± 0.21 b	5.36 ± 0.18 c	-
UFA	86.75 ± 2.32 a	84.74 ± 1.65 a	84.46 ± 0.53 a	84.63 ± 0.79 a	-
SFA	14.33 ± 0.85 a	14.79 ± 0.73 a	14.39 ± 0.33 a	15.94 ± 1.66 a	-
MUFA/PUFA	7.50 ± 0.52 c	5.95 ± 0.04 d	10.43 ± 0.25 b	14.91 ± 0.36 a	-

* C16:0—palmitic acid; C16:1—palmitoleic acid; C17:0—heptadecanoic acid; C17:1—heptadecenoic acid; C18:0—stearic acid; C18:1—oleic acid; C18:2—linoleic acid; C18:3—linolenic acid; C 20:0—arachidonic acid; C20:1—gadoleic acid; C22:0—behenic acid; C24:0—lignoceric acid. MUFA—monounsaturated fatty acid; PUFA—polyunsaturated fatty acid; UFA—unsaturated fatty acid; SFA—saturated fatty acid. Results are expressed as mean values of three repetitions ± standard deviation (SD). * Actual limits for extra virgin olive oil category [4]. The means within each row, labeled by different letters, are significantly different (Tukey’s test, *p* ≤ 0.05).

**Table 3:** Volatile composition of virgin olive oils obtained from Dalmatian autochthonous cultivars (identification and mean proportion values of peak area) detected by HS-SPME/GC-MS.

				Oblica	Lastovka	Levantinka	Krvavica
Compound	Identification Method	KI *	KI-Ref	Mean * (%)	Mean (%)	Mean (%)	Mean (%)
				±SD	±SD	±SD	±SD
***Aldehydes***							
Hexanal	KI, MS, RT	1072	1074 ^1^, 1086 ^2^, 1073 ^3^	12.74 ± 4.59 b	23.69 ± 2.87 a	1.34 ± 1.05 c	13.76 ± 0.04 b
*Z*-3-Hexenal	KI, MS	1135	1137 ^1^, 1115 ^2^	2.87 ± 3.55 a	0.48 ± 0.01 a	0.25 ± 0.28 a	5.91 ± 1.61 a
Heptanal	KI, MS	1191	1184 ^1^, 1190 ^2^	0.04 ± 0.02 b	0.39 ± 0.03 a	0.03 ± 0.02 b	0.16 ± 0.07 b
*E*-2-Hexenal	KI, MS, RT	1208	1216 ^1^, 1225 ^2^, 1129 ^3^	48.03 ± 9.66 a	43.3 ± 16.92 a	74.84 ± 1.48 b	45.28 ± 1.44 a
(*E*,*E*) or (*E*,*Z*)-2,4-Hexadienal	KI, MS	1384	1397 ^1^, 1402 ^3^	1.45 ± 1.42 a	0.26 ± 0.02 a	0.30 ± 0.35 a	1.55 ± 1.83 a
(*E*,*E*) or (*E*,*Z*)-2,4-Hexadienal	KI, MS	1388	1397 ^1^, 1402 ^3^	8.23 ± 8.48 a	4.15 ± 0.5 a	1.84 ± 2.45 a	13.36 ± 2.67 a
*(Z)*-2-Heptenal	KI, MS	1314	1320 ^4^	0.08 ± 0.07 b	1.08 ± 0.1 a	0.10 ± 0.04 b	0.18 ± 0.1 b
Octanal	KI, MS, RT	1282	1288 ^1^, 1296 ^2^, 1297 ^3^	0.05 ± 0.02 a	7.50 ± 8.72 a	0.05 ± 0.02 a	0.22 ± 0.11 a
*E*,*E*-2,4- Heptadienal	KI, MS	1451	1463 ^1^	0.18 ± 0.18 a	0.44 ± 0.27 a	0.22 ± 0.23 a	0.54 ± 0.03 a
*Total aldehydes*				73.67 ± 8.68 a	81.29 ± 6.19 a	78.97 ± 0.85 a	80.94 ± 4.32 a
***Alcohols***							
1-Penten-3-ol	KI, MS	1159	1164 ^1^, 1166 ^2^, 1163 ^3^	1.61 ± 1.26 a	2.42 ± 2.55 a	0.51 ± 0.16 a	2.12 ± 0.17 a
*Z*-2-Penten-1-ol	KI, MS, RT	1302	1320 ^1^, 1329 ^2^, 1321 ^3^	0.90 ± 0.88 a	2.44 ± 0.61 a	0.73 ± 0.41 a	1.88 ± 2.21 a
*E*-2-Penten-1-ol	KI, MS	1310	1320 ^2^, 1333 ^3^	2.40 ± 1.48 b	6.22 ± 0.06 a	0.84 ± 0.38 b	2.74 ± 0.41 b
Hexanol	KI, MS, RT	1344	1357 ^1^, 1362 ^2^, 1360 ^3^, 1354 ^4^	0.20 ± 0.10 b	1.01 ± 0.19 b	4.49 ± 1.12 a	0.40 ± 0.09 b
*E*-3-Hexen-1-ol	KI, MS, RT	1354	1366 ^1^, 1372 ^2^, 1372 ^3^	0.15 ± 0.01 a	0.04 ± 0 b	0.20 ± 0.05 a	0.10 ± 0.01 ab
*Z*-3-Hexen-1-ol	KI, MS, RT	1374	1385 ^1^, 1392 ^2^, 1385 ^3^, 1388 ^4^	13.25 ± 6.33 a	1.24 ± 0.27 a	7.35 ± 0.98 a	2.97 ± 1.37 a
*Total alcohols*				18.51 ± 2.62 a	13.37 ± 3.19 a	14.12 ± 1.91 a	10.21 ± 0.16 a
***Esters***							
Methyl acetate	KI, MS	<1000	800 ^1^	0.4 ± 0.07 a	0.34 ± 0.02 a	0.29 ± 0.26 a	0.27 ± 0.04 a
Ethyl acetate	KI, MS, RT	<1000	892 ^1^, 895 ^3^	0.06 ± 0.02 a	0.78 ± 0.79 a	0.49 ± 0.52 a	0.11 ± 0.01 a
Hexyl acetate	KI, MS, RT	1268	1247 ^1^, 1281 ^2^, 1269 ^4^	0.00 ± 0.00 b	0.63 ± 0.02 a	0.08 ± 0.08 b	0.05 ± 0.00 b
*Total esters*				0.46 ± 0.05 a	1.75 ± 0.80 a	0.86 ± 0.70 a	0.43 ± 0.03 a
***Terpenes***							
α-Copaene	KI, MS	1487	1481 ^1^, 1500 ^2^, 1505 ^3^	1.30 ± 1.53 a	0.34 ± 0.17 a	2.10 ± 1.58a	3.15 ± 1.54 a
*Total terpenes*				1.30 ± 1.53 a	0.34 ± 0.17 a	2.10 ± 1.58a	3.15 ± 1.54 a
***Organic acids***							
Acetic acid	KI, MS, RT	1430	1448 ^1^	1.17 ± 1.18 a	2.67 ± 3.66 a	0.03 ± 0.03 a	4.84 ± 5.59 a
Propanoic acid	KI, MS	1519	1528 ^1^	0.06 ± 0.04 a	0.14 ± 0.04 a	0.13 ± 0.11 a	0.27 ± 0.04 a
*Total organic acids*				1.23 ± 1.14 a	2.81 ± 3.62 a	0.16 ± 0.14 a	5.11 ± 5.64 a

* Results are expressed as mean values of three independent repetitions ± standard deviation. The means within each row, labeled by different letters, are significantly different (Tukey’s test, *p* ≤ 0.05). Identification methods: RT—identification by comparison with retention times and mass spectra of pure standards; MS—identification by comparison with mass spectra from the NIST05 library; KI—identification by comparison with Kováts’ retention indexes from the literature (KIref) (^1^ [38]; ^2^ [68]; ^3^ [16]; ^4^ [48]). KI*—Kováts’ retention indexes on Rtx-WAX capillary columns.

## Data Availability

Not applicable.
